# Topotactic Oxidation of Perovskites to Novel SrMo_1-x_M_x_O_4−δ_ (M = Fe and Cr) Deficient Scheelite-Type Oxides

**DOI:** 10.3390/ma13194441

**Published:** 2020-10-06

**Authors:** V. Cascos, R. Martínez-Coronado, M. T. Fernández-Díaz, J. A. Alonso

**Affiliations:** 1 Instituto de Ciencia de Materiales de Madrid, Consejo Superior de Investigaciones Científicas, Cantoblanco, E-28049 Madrid, Spain; rmartinezcoronado@gmail.com (R.M.-C.); jaalonso@icmm.csic.es (J.A.A.); 2 Departamento de Química Inorgánica, Universidad Complutense de Madrid, E-28040 Madrid, Spain; 3 Institut Laue Langevin, BP 156X, F-38042 Grenoble, France; ferndiaz@ill.fr

**Keywords:** scheelites, SrMoO_4_, oxygen-vacancy, reversibility, SrMoO_3_, SOFC, solid oxide fuel cells, anode material

## Abstract

New polycrystalline SrMo_1−x_M_x_O_4−δ_ (M = Fe and Cr) scheelite oxides have been prepared by topotactical oxidation, by annealing in air at 500 °C, from precursor perovskites with the stoichiometry SrMo_1−x_M_x_O_3−δ_ (M = Fe and Cr). An excellent reversibility between the oxidized Sr(Mo,M)O_4−δ_ scheelite and the reduced Sr(Mo,M)O_3−δ_ perovskite phase accounts for the excellent behavior of the latter as anode material in solid-oxide fuel cells. A characterization by X-ray powder diffraction (XRD) and neutron powder diffraction (NPD) has been carried out to determine the crystal structure features. The scheelite oxides are tetragonal, space group *I4_1_/a* (No. 88). The Rietveld-refinement from NPD data at room temperature shows evidence of oxygen vacancies in the structure, due to the introduction of Fe^3+^/Cr^4+^ cations in the tetrahedrally-coordinated B sublattice, where Mo is hexavalent. A thermal analysis of the reduced perovskite (SrMo_1−x_M_x_O_3−δ_) in oxidizing conditions confirms the oxygen stoichiometry obtained by NPD data; the stability range of the doped oxides, below 400–450 °C, is lower than that for the parent SrMoO_3_ oxide. The presence of a Mo^4+^/Mo^5+^ mixed valence in the reduced SrMo_1−x_M_x_O_3−δ_ perovskite oxides confers greater instability against oxidation compared with the parent oxide. Finally, an XPS study confirms the surface oxidation states of Mo, Fe, and Cr in the oxidized samples SrMo_0.9_Fe_0.1_O_4-δ_ and SrMo_0.8_Cr_0.2_O_4-δ_.

## 1. Introduction

Solid oxide fuel cells (SOFCs) are electrochemical devices that can offer clean and efficient power generation to almost any electrical power device in the world [1]. In this field, new SrMo_1−x_M_x_O_3−δ_ (M = Fe and Cr; *x* = 0.1 and 0.2) perovskite materials have been recently prepared and characterized and perform successfully as anodes for solid-oxide fuel cells (SOFC) [2,3,4,5]. The mixed electronic and ionic conductivity (MIEC) induced in these phases creates oxygen vacancies at the working temperatures of SOFC (700–850 °C). This MIEC behavior results from the combination of the excellent metallic conductivity of the pristine SrMoO_3_ oxide [6,7] together with the effect of doping with trivalent elements (M = Fe, Cr) at the Mo^4+^ position. These perovskite phases are stable in reducing conditions (H_2_ atmosphere), as required for anode materials [8,9,10,11]; however, the annealing of the perovskite oxides in air leads, reversibly, to scheelite-like oxides. The reversibility of the scheelite (oxidized) and perovskite (reduced) oxides is required for the cycling and performance of the fuel cells.

In recent years, scheelite-structured oxides, represented by the generic form *AB*O_4_ (where *A* = Ca, Sr, Ba, Pb, or Cd; *B* = Mo or W), have found application in different technological fields due to their remarkable luminescence and appealing structural properties [12,13,14,15]. Metal molybdates (*A*MoO_4_) of relatively large divalent cations (ionic radius >0.99 Å) crystallize in the scheelite structure (e.g., CaWO_4_) [16,17,18,19,20], defined in the tetragonal *I*4_1_*/a* space group with four formula units per unit cell. The voluminous A cations are located at the 4b Wyckoff positions, with the coordination sphere of eight oxygen atoms in the shape of a trigonal dodecahedron. The B atoms form tetrahedral [BO_4_]^2−^ entities, with the central B^6+^ cations placed at 4a positions.

Scheelite materials can be related to a fluorite structure and possess a rather open framework, in which A and B cations are long-range ordered. In a scheelite phase, the cation sublattice forms a face-centered cubic (FCC) array, and the anions are located in tetrahedrally coordinated interstitial positions [21]. A distortion of the anion lattice is due to the difference in cation sizes. The larger A cations display eight-fold coordination (four near, and four more distant), and the smaller B cation is four-fold (tetrahedrally) coordinated. Alternatively, scheelite can also be defined by corner sharing chains of alternating [MoO_4_] tetrahedra and [AO_8_] bisdisphenoid units along the *a*-axis of the structure [22].

In this work, the preparation and characterization of two new families of oxygen-deficient scheelite oxides with formula SrMo_1−*x*_M*_x_*O_4−δ_ (M = Fe and Cr; *x* = 0.1 and 0.2) resulting from the topotactical oxidation of the corresponding perovskite oxides is described. The materials have been characterized by neutron powder diffraction (NPD) to determine their crystallographic features, such as the presence of oxygen vacancies and the amount of M atoms in the B sublattice. To complete the study, thermal analysis and XPS analysis were carried out to evaluate the stability of the doped compounds and their reversibility and to find out the surface oxidation states of the different elements in the scheelite-type materials. The transformation between the perovskite (reduced) and scheelite (oxidized) structures, at relatively low temperatures, in topotactic conditions involving shifts of a few atoms within an almost unchanged framework, is discussed.

## 2. Experimental Procedures

SrMo_1−*x*_M*_x_*O_4−δ_ (M = Fe and Cr, *x* = 0.1 and 0.2) scheelites were synthesized by the citrate method described in References [1,2,3]. Stoichiometric quantities of Sr(NO_3_)_2_, (NH_4_)_6_Mo_7_O_24_·4H_2_O, C_2_FeO_4_·2H_2_O, or r(NO_3_)_3_·9H_2_O were dissolved in citric acid with some drops of nitric acid. The resins were decomposed at 600 °C for 12 h in air, which produced pure scheelite oxide phases with a moderate crystallinity. These scheelite precursor powders were then reduced in forming gas (5% H_2_/95% N_2_) flow at 1100 °C for 12 h to obtain pure perovskite SrMo_1-x_M_x_O_3-δ_ phases. Finally, the perovskite materials were reoxidized at 500 °C in air for 1 h to obtain well-crystallized scheelite oxides, enabling a detailed structural study to be conducted.

SrMo_1−*x*_M*_x_*O_4−δ_ (M = Fe and Cr) scheelites were first analyzed by XRD with a Brucker D8 diffractometer with Cu Kα radiation (λ = 1.5418 Å) to check the purity of the materials. A nickel filter allows the complete removal of Cu K_β_ radiation. An in-depth study of two selected scheelite samples with M = Fe, *x* = 0.1 and M = Cr, *x* = 0.2 was carried out by NPD at the D2B diffractometer of the Institut Laue-Langevin, (Grenoble, France), with a wavelength λ = 1.590 Å, at 25 °C. About 2 g of the sample were contained in a vanadium can; the collection time for each diffractogram was 2 h. The diffractograms were analyzed by the Rietveld method [23], using the FULLPROF refinement program, version December 2008 [24]. A pseudo-Voigt function generated the profile shape. Positional coordinates, scale factor, background coefficients, zero-point error, isotropic displacement for all the atoms, and occupancy factors for oxygen atoms were refined in the final run. The coherent scattering lengths for Sr, Fe, Cr, Mo, and O were 7.02, 9.45, 3.635, 6.72, and 5.803 fm, respectively.

The thermogravimetric profiles were performed in a Mettler TA3000 system equipped with a TC10 processor unit (Mettler Toledo, Madrid, Spain). Thermogravimetric curves (TGA) were measure in a TG50 microbalance, working at a heating rate of 10 °C min^−1^, in an oxidizing atmosphere (oxygen O_2_ flow) of 0.3 L min^−1^. About 50 mg of the “reduced” perovskite samples were used as starting materials in each experiment. The differential thermal analysis (DTA) was performed for SrMo_0.8_Cr_0.2_O_3-δ_ in an SDT Q600 V20.9 Build 20 thermal gravimetric equipment in the same conditions as the TG analysis. About 86 mg of the SrMo_0.8_Cr_0.2_O_3−δ_ perovskite was used in this experiment.

Photoelectron spectra of two selected oxidized phases were recorded on a SPECS GmbH electron (Madrid, Spain) spectrometer equipped with a hemispherical electron analyzer, using a MgKα (hν = 1253.6 eV, 1 eV = 1.603 × 10^–19^ J) X-ray source (200 W, 12 kV). After degassing at 10^−6^ mbar, the samples were transferred to the ion-pumped analysis chamber, where the residual pressure was kept below 4 × 10^−9^ mbar during the data acquisition. All binding energies (BE) were referenced to the C 1s signal as an internal standard at 284.8 eV from carbon contamination of the samples to correct the charging effects. Peak intensities were estimated by calculating the integral of each peak after subtracting an S-shaped background and fitting the experimental peak to a combination of Lorentzian/Gaussian lines of variable proportions. Atomic surface contents were estimated from the areas of the peaks, corrected using the corresponding sensitivity factors [25].

## 3. Results and Discussion

### 3.1. Crystallographic Characterization

**Figure 1:**
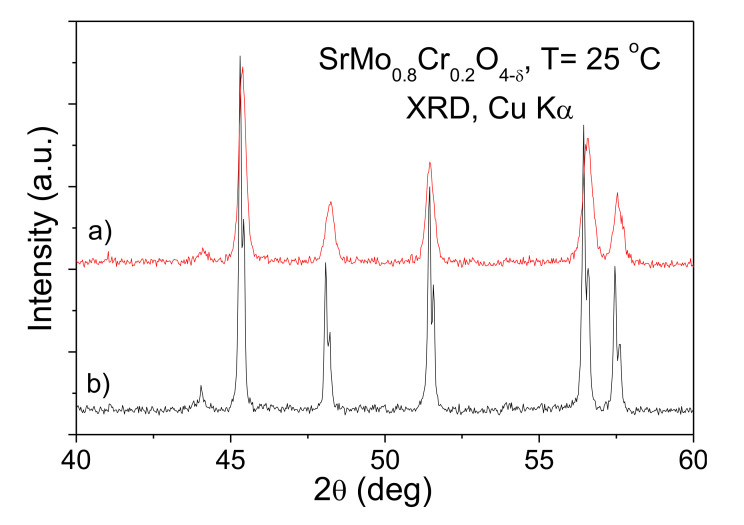
XRD patterns for SrMo_0.8_Cr_0.2_O_4−δ_ (**a**) by direct treatment in air of the citrate precursors, (**b**) by reoxidizing topotactically at 500 °C in air, of the reduced perovskites.

The SrMo_1−*x*_M*_x_*O_4−δ_ (M = Fe and Cr, *x* = 0.1 and 0.2) oxides obtained by direct treatment in air of the citrate precursors were single-phase scheelites with poor crystallinity, as illustrated in Figure 1a for M = Cr, *x* = 0.2. Once the scheelites are reduced to perovskites (1100 °C in 5% H_2_) and finally reoxidized topotactically at 500 °C in air, a much better crystallinity is observed (Figure 1b), although the unit-cell and other crystallographic parameters are unchanged. The same results are observed for all the samples; no impurity phases were detected in the XRD patterns (they are all shown in the Supplementary Material, Figure S1).

Table S1 (Supplementary Information) shows the unit-cell parameters determined by XRD for all the samples.

**Figure 2:**
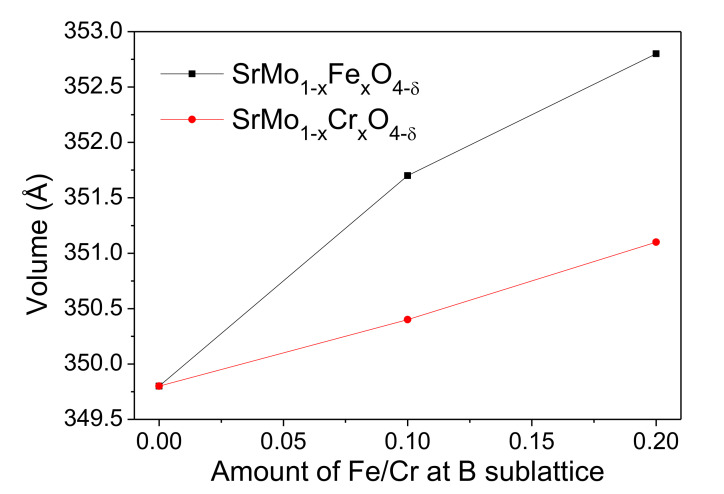
Variation of the unit-cell volume as a function of the M-doping content, from XRD data.

Figure 2 shows the evolution of the volume of the samples as the doping level increases for Fe and Cr. A significant expansion of the volume is observed as Mo is replaced by M (M = Fe and Cr). For instance, the volume of SrMoO_4_ is 349.8(2) Å^3^ [26], and for SrMo_0.9_Fe_0.1_O_4−δ_ and SrMo_0.8_Fe_0.2_O_4−δ_ the volumes are 351.75(1) Å^3^ and 352.87(2) Å^3^, respectively; the same result is observed for Cr doped samples, but with smaller values. The expansion of the volume is a result of the greater ionic radius of ^IV^Fe^3+^ (0.49 Å) compared to that of ^IV^Mo^6+^ (0.41 Å) in four-fold coordination [27]. In the case of Cr, the analysis of the neutron data combined with the TGA results (Section 3.3.) strongly suggests that Cr is oxidized from Cr^3+^ in the perovskite structure to Cr^4+^ in the scheelite network, upon adopting four-fold coordination. The tabulated ionic radius for ^IV^Cr^4+^ (0.41 Å) is analogous to that of Mo^6+^; in this case, the moderate expansion of the cell upon Cr doping with respect to SrMoO_4_ must be accounted by the simultaneous presence of a significant number of oxygen vacancies, which relax the chemical bonds across the crystal and drive an increase of the unit-cell parameters.

An NPD study at room temperature (RT) of two selected Fe (*x* = 0.1) and Cr (*x* = 0.2) doped samples was carried out to investigate structural details. The crystal structure was defined in the tetragonal *I4_1_/a* (No 88) space group, Z = 4. Sr atoms are located at 4*b* (0, 1/4, 5/8), Mo and M (M = Fe and Cr) are distributed at random at 4*a* (0, 1/4, 1/8), and oxygen atoms O1 at 16*f* (x, y, z) sites. The occupancy factors of oxygen atoms were also refined in the final run; the introduction of M ions at the Mo^6+^ sublattice is effective in the creation of oxygen vacancies, giving rise to an oxygen-deficient scheelite. The refined occupancy factors of oxygen (O) and the Mo/M ratio for the doped samples lead to the final SrMo_0.88(1)_Fe_0.12(1)_O_3.77(3)_ and SrMo_0.78(1)_Cr_0.22(1)_O_3.85(2)_ stoichiometries. The oxidation states of Mo, Fe, and Cr are discussed in the next sub-section in combination with the TGA data.

**Figure 3:**
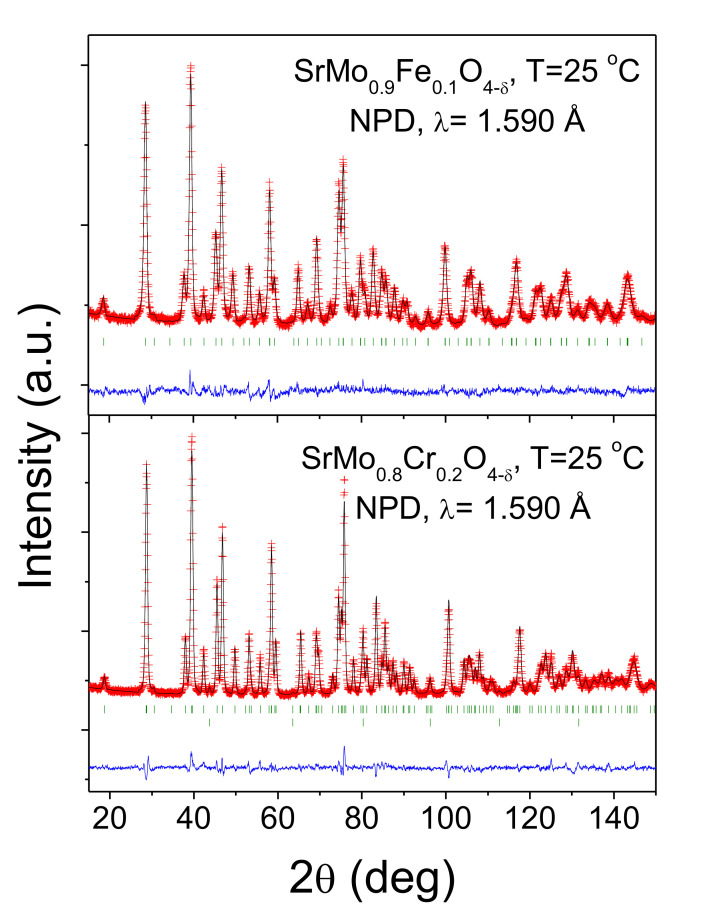
Observed (crosses), calculated (full line), and difference (at the bottom) neutron powder diffraction (NPD) profiles for SrMo_0.9_Fe_0.1_O_4−δ_ and SrMo_0.8_Cr_0.2_O_4−δ_ at 25 °C, refined in the tetragonal *I4_1_/a* space group. The vertical markers correspond to the allowed Bragg reflections. The second series of Bragg reflections correspond to vanadium from the sample holder.

Figure 3 illustrates the good agreement between the observed and calculated NPD patterns for nominal SrMo_0.9_Fe_0.1_O_4−δ_ and SrMo_0.8_Cr_0.2_O_4−δ_ oxides at room temperature. Table 1 summarizes the unit-cell, atomic, displacement parameters, and the discrepancy factors, after the Rietveld refinements of the doped samples at room temperature.

Table 2 contains the main interatomic distances and angles. The (Sr–O1) bond lengths at room temperature for the doped samples compare reasonably well with the expected values calculated as sums of the ionic radii, displaying 2.592 Å versus the calculated 2.660 Å (^VIII^Sr^2+^ = 1.26 Å) [27]. The tetrahedral group around Mo for the parent SrMoO_4_ exhibits four Mo–O bonds at 1.767 Å [26]. In the doped samples, the introduction of cations of larger ionic size leads to a stretching of the tetrahedral group (Mo,M) with four (Mo,M)–O bonds at 1.8089(4) Å for SrMo_0.9_Fe_0.1_O_4−δ,_ and 1.7892(3) Å for SrMo_0.8_Cr_0.2_O_4−δ_.

The parent SrMoO_4_ shows a very slight distortion of the MoO_4_ tetrahedral units (four angles of (α) 107.36° and two of (β) 113.14° [26]); the same distortion is observed in the doped samples, although the distortion is slightly bigger: e.g., (α) 107.87° and (β) 112.71° for Cr with x = 0.2.

### 3.2. Thermal Analysis (TGA)

**Figure 4:**
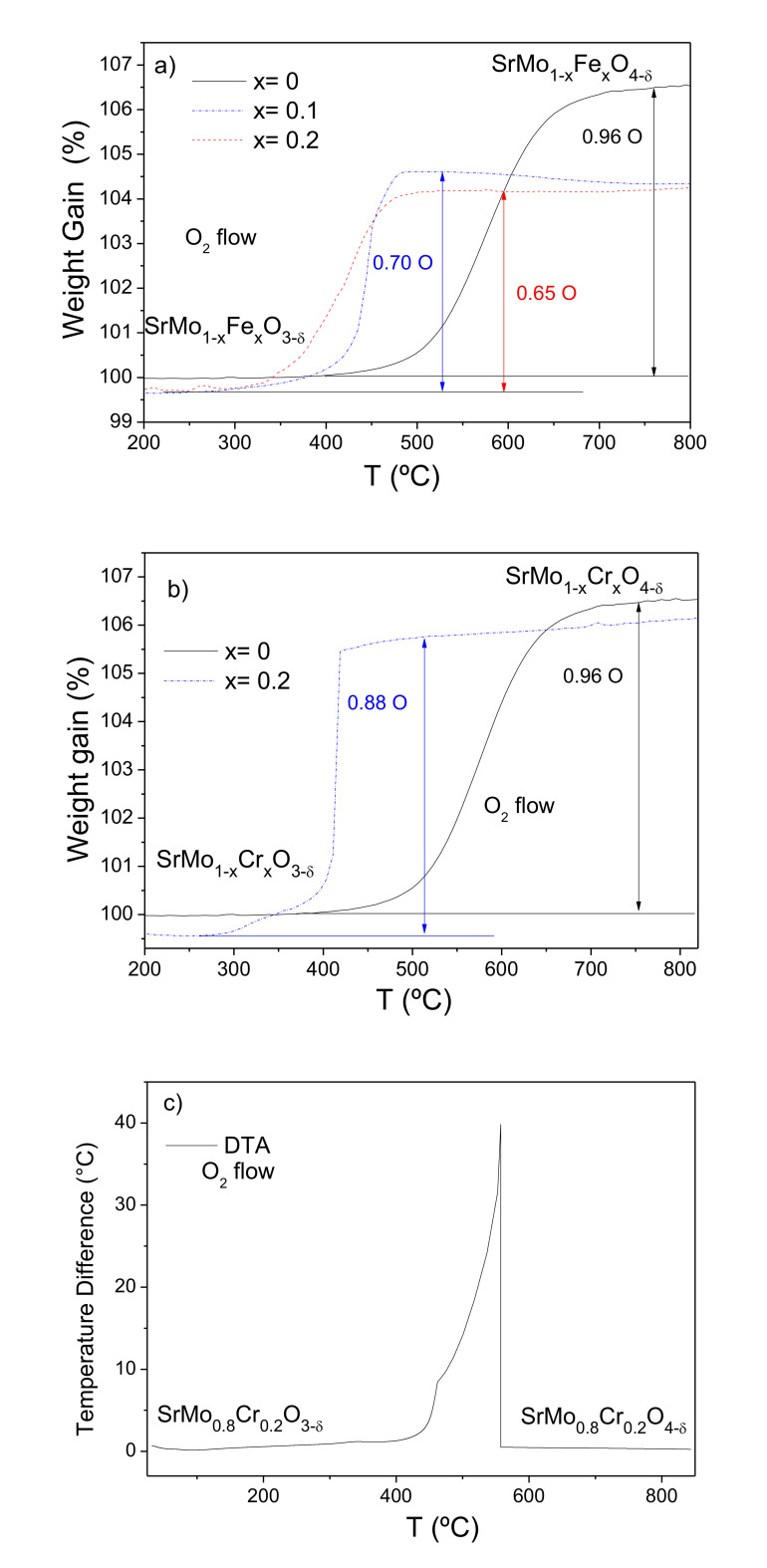
Thermal analysis in O_2_ flow (TG curve) of (**a**) SrMo_1−*x*_Fe*_x_*O_3−δ_, (**b**) SrMo_1−*x*_Cr*_x_*O_3−δ_ perovskites, showing an oxidation step of the oxygen-deficient SrMo_1−*x*_M*_x_*O_4−δ_ scheelite phase and (**c**) differential thermal analysis (DTA) of SrMo_0.8_Cr_0.2_O_3−δ_ perovskite.

The thermal evolution of the doped samples was studied by recording TGA curves. Heating the “reduced” perovskite phases SrMo_1−x_M_x_O_3−δ_ (M = Fe and Cr, x = 0, 0.1 and 0.2) in an oxygen atmosphere leads to the oxidation of these materials to give the SrMo_1−x_M_x_O_4−δ_ scheelite structures. Figure 4a,b, for Fe and Cr, respectively, show the thermal analysis curves obtained in O_2_, displaying the incorporation of oxygen atoms in the 400–450 °C temperature range for all the doped samples. The incorporation of oxygen atoms occurs at lower temperatures and in a more abrupt way for the M-doped samples, especially for M = Cr, compared with the parent SrMoO_3_ oxide. For SrMoO_3_, the incorporation of oxygen is 0.96 atoms per formula unit, very close to the expected amount to give the SrMoO_4_ scheelite. For M = Fe, the samples uptake 0.70 and 0.65 oxygens per formula unit for x = 0.1 and 0.2, respectively. A previous NPD study of the reduced perovskites [2] demonstrated that, at room temperature, the oxygen sublattice is virtually complete, within the standard deviations, e.g., SrMo_0.9_Fe_0.1_O_2.988(2)_ and SrMo_0.8_Fe_0.2_O_2.992(9)_ at 25 °C, in which the oxidation states for Mo are 4.08+ and 4.23+ for x = 0.1 and 0.2, respectively. The stoichiometry for the oxidized scheelites from the TGA weight is SrMo_0.9_Fe_0.1_O_3.70_ and SrMo_0.8_Fe_0.2_O_3.65_. The NPD data for the x = 0.1 sample lead to a close oxygen stoichiometry of 3.77(3). Assuming that Fe is trivalent during the process, we obtain oxidation states for Mo of 5.67+ and 5.88+ for *x* = 0.1 and 0.2, respectively. Taking into account that even for the parent SrMoO_3_ compound, the oxidation to SrMoO_4_ under the same conditions yields Mo^5.92+^, these data would suggest that in all cases, Mo is hexavalent, although a small proportion of Mo^5+^ is not to be discounted.

The case of M = Cr is totally different. The oxygen uptake is significantly greater for the *x* = 0.2 composition with 0.88 oxygens per formula unit. The assumption that Cr remains in its trivalent oxidation state would lead to Mo valences considerably greater than 6+, which is not chemically consistent. We suggest that in this case, Cr^3+^ present in the reduced perovskite phases becomes oxidized to an admixture of Cr^4+^–Cr^5+^ions, adopting a tetrahedral oxygen coordination in the scheelite structures. Cr^4+^ is well known to exhibit tetrahedral coordination in many chromates, e.g., Sr_2_CrO_4_ [28], and Cr^5+^ has also been described in tetrahedral coordination in compounds isostructural with silicates and phosphates, for instance, YCrO_4_ with zircon structure [28]. The oxidation of SrMo_0.8_Cr_0.2_O_3_ perovskite leads to a scheelite phase of stoichiometry SrMo_0.8_Cr_0.2_O_3.88_, which compares well with the determined stoichiometry from NPD data of SrMo_0.78_Cr_0.22_O_3.85(2)_. Assuming hexavalent Mo in this case, we obtain a Cr valence of 4.64+ for Cr from the neutron determination or 4.8+ from TGA data. XPS measurements (described below) indicate Cr^6+^ at the surface; this is compatible with our TGA results for bulk samples since it is reasonable that Cr is oxidized beyond these oxidation states to Cr^6+^ in the first atomic layers, given that Cr^4+^ and Cr^5+^ are highly unstable cations. Cr^6+^ is also well known to exhibit tetrahedral coordination in many chromates, e.g., K_2_Cr_2_O_7_ [28,29]. The oxidation of Cr^3+^ to superior oxidation states is perhaps related to the abrupt increase of weight observed in the TGA curves, and the smaller oxidation temperatures (starting below 400 °C) compared with the Fe-doped compounds.

Figure 4c displays the differential thermal analysis (DTA) curve of SrMo_0.8_Cr_0.2_O_3−δ_ perovskite showing an exothermic peak between 400 °C and 550 °C that matches with the oxidation process observed by the TG analysis, thus confirming that this sample becomes a scheelite material around 550 °C.

Finally, a thermal treatment of the scheelite phases in reducing (H_2_ (5%)/N_2_) conditions restores the perovskite phases, exhibiting excellent reversibility.

### 3.3. XPS Measurements

**Figure 5:**
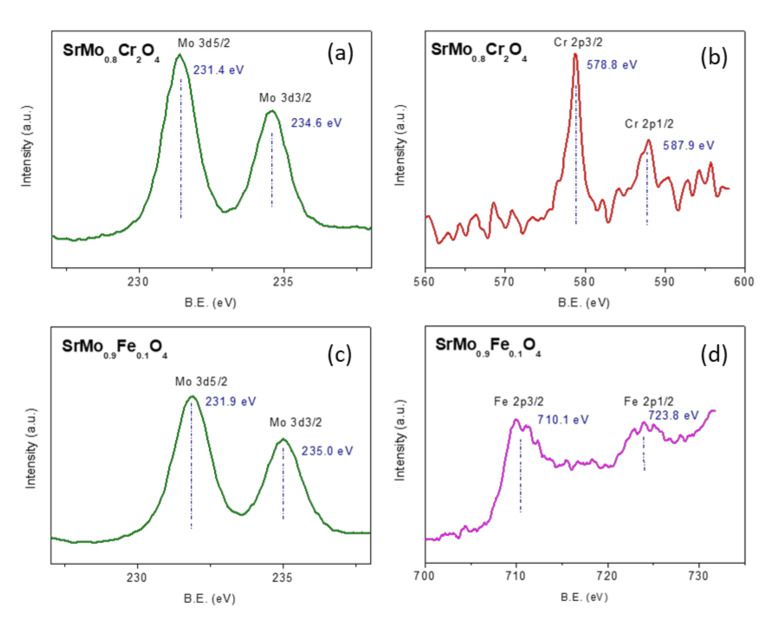
XPS core level spectra of Mo 3d (**a**) and Cr 2p (**b**) of SrMo_0.8_Cr_0.2_O_4,_ and Mo 3d (**c**) and Fe 2p (**d**) of SrMo_0.9_Fe_0.1_O_4._

The XPS technique was used for determining the surface oxidation states of Mo, Fe, and Cr in the samples SrMo_0.9_Fe_0.1_O_4−δ_ and SrMo_0.8_Cr_0.2_O_4−δ_. The XPS spectra of Mo 3d, Cr 2p, and Fe 2p core levels are depicted in Figure 5. As observed, the core binding energy values for Mo 3d5/2 level (234.6–235.0 eV) in both samples reflect the stabilization of the 6^+^ oxidation state for Mo. The splitting between Mo 3d5/2 and Mo 3d3/2 core levels is 3.2 eV for SrMo_0.8_Cr_0.2_O_4_, which is characteristic of Mo^6+^. In the case of the sample SrMo_0.9_Fe_0.1_O_4_, the splitting is slightly lower, suggesting the presence of a very small proportion of Mo^5+^ on the surface of this sample. The Cr 2p3/2 level shows a binding energy of 578.8 eV, ascribed to Cr^6+^ surface species. Concerning the oxidation state of Fe species on the surface of SrMo_0.9_Fe_0.1_O_4_, the results derived from the analysis of Fe 2p3/2 and Fe 2p1/2 levels, with core-binding energies of 710.1 and 723.8, respectively, (with a splitting of 13.6 eV) are related with surface species of Fe^3+^ [30]. These results are consistent with those obtained by X-ray and neutron diffraction and by TGA, confirming that surface species of Cr are mainly as Cr^6+^.

### 3.4. Topotactical Oxidation

**Figure 6:**
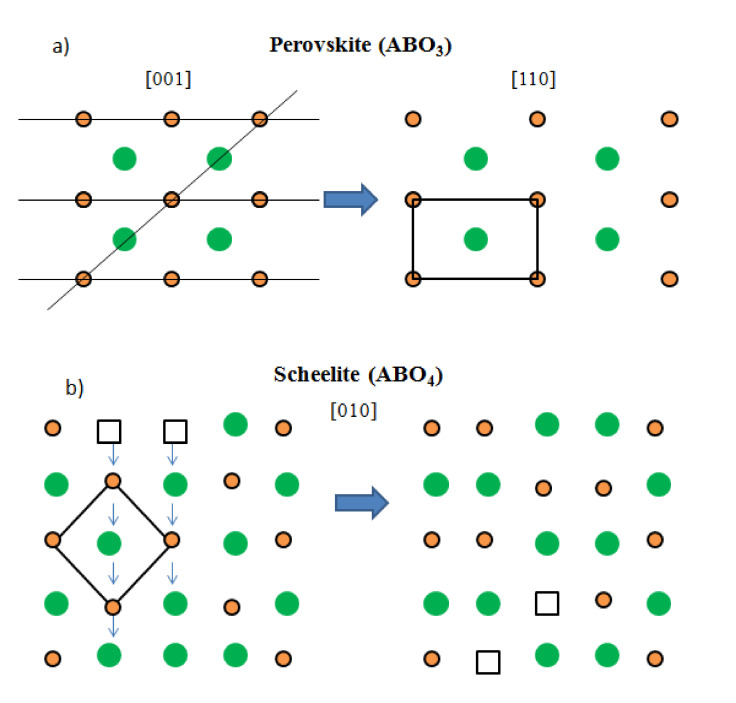
Topotactic transformation from the (**a**) perovskite structure to the (**b**) scheelite structure at 600 °C. The large green spheres represent Sr atoms; small red circles are Mo atoms; squares represent Sr vacancies. The shift of double rows of Sr atoms via vacancies accounts for the transformation of the metal substructures. The rearrangement of oxygen atoms is detailed in Figure 7.

In principle, it may seem surprising that the “reduced” perovskite structure is transformed into an “oxidized” scheelite network by topotactical insertion of oxygen atoms, at moderate temperatures as low as 400 °C, where the mobility of cationic species is very limited. This can only be explained because of the topological relationship existing between both structural types. Figure 6a shows the crystal structure of the Sr(Mo, M)O_3_ perovskite aristotype (undistorted cubic unit cell with *a*_0_ ≈ 3.95 Å, defined in the *Pm-3m* space group), where the Sr cations are surrounded by 12 oxygens in dodecahedron coordination; (Mo,M) cations exhibit an octahedral coordination, surrounded by six oxygen atoms. Looking at the distribution of the metal ions, a (1:1:0) plane shows a rectangular checkerboard-like arrangement where Sr and (Mo,M) alternate in the two directions.

**Figure 7:**
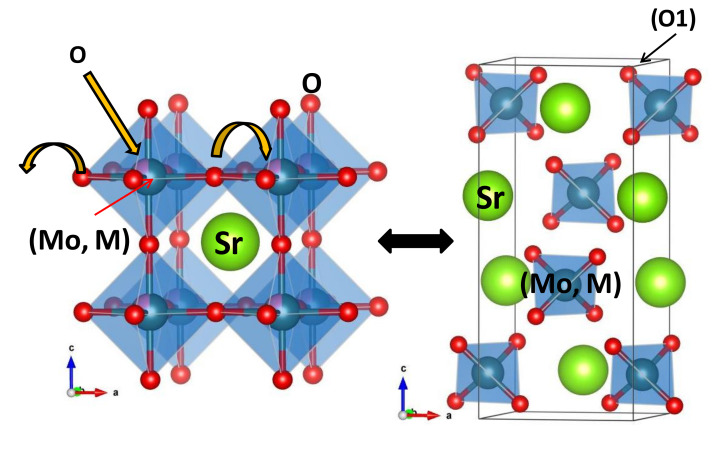
Perovskite and scheelite structures for SrMo_1−x_M_x_O_4−δ_ (M = Fe and Cr).

Figure 6b shows a projection of the scheelite structure on the (0:1:0) plane, highlighting the arrangement of the metal ions as dimer units of A A and B B atoms that also exhibit a checkerboard-like distribution. Figure 6 concerns the metal sublattice, and it shows that the perovskite metal network can be simply transformed into the scheelite arrangement by shifting double rows of metal atoms, which can happen by means of point defects (metal vacancies) at moderate temperatures. The octahedral oxygen coordination BO_6/2_, sharing corners at the perovskite structure, is easily transformed into the tetrahedral environment BO_4_ in isolated units, required in the scheelite phase, by shifts of the oxygen atoms, combined with the topotactical insertion of one more oxygen per formula unit. This is illustrated in Figure 7, where each B atom of the perovskite keeps three oxygen atoms and accepts one more from the atmosphere to give the BO_4_ units, forming dimmers, as mentioned before. The existence of a large number of point defects (metal vacancies) across the solid seems essential to account for the mobility and shifts of rows of metal atoms at moderate temperatures.

## 4. Conclusions

In summary, we prepared a new family of scheelite oxides, with composition SrMo_1−x_M_x_O_4−δ_, by topotactical oxidation of precursor perovskites of stoichiometry SrMo_1−x_M_x_O_3−δ_. The crystal structure was refined at room temperature in the tetragonal *I4_1_/a* space group from NPD data, providing evidence for the presence of oxygen vacancies in the structure. The introduction of M cations in the B sublattice, where Mo is hexavalent, led to oxygen-deficient scheelite structures. Whereas Fe was trivalent in both perovskite and scheelite phases, Cr^3+^ was oxidized to Cr^4+^ and beyond upon the perovskite to scheelite structural transformation at moderate temperatures. Thermogravimetric analysis (TGA) in oxidizing conditions confirmed both the oxygen stoichiometries obtained by NPD data and the lower stability range of the perovskite oxides in O_2_ atmosphere, below 400 °C–450 °C, compared to the parent SrMoO_4_ oxide. The presence of a Mo^4+^/Mo^5+^ mixed valence in the perovskite oxides, as well as the abrupt oxidation of Cr^3+^ to Cr^4+^ conferred greater instability against oxidation compared with the parent oxide. A DTA analysis confirmed that an abrupt oxidation process occurred between 400 °C and 550 °C. XPS analysis unveiled a mixed Mo^5+^–Mo^6+^ valence for the Fe scheelite, whereas Cr^6+^ was identified at the surface for the Cr scheelite. In addition, an excellent reversibility between the oxidized Sr(Mo,M)O_4−δ_ scheelite and the reduced Sr(Mo,M)O_3-δ_ perovskite phases was observed. The transformation between perovskite and scheelite networks by topotactical insertion of oxygen atoms, at moderate temperatures as low as 400 °C, where the mobility of cationic species is very limited, can only be explained because of the topological relationship existing between both structural types.

## Figures and Tables

**Table 1:** Unit-cell, displacement parameters, interatomic distances, and angles for SrMo_1-*x*_M*_x_*O_4−δ_ in tetragonal *I4_1_/a* (No 88) space group, Z = 4, from NPD at 25 °C. Sr is placed at 4*b* (0, 1/4, 5/8); Mo, M at 4*a* (0, 1/4, 1/8), and O at 16*f* (x,y,z) sites.

	SrMo_0.9_Fe_0.1_O_4−δ_	SrMo_0.8_Cr_0.2_O_4−δ_
a (Å)	5.3997(2)	5.3735(2)
b (Å)	5.3997(2)	5.3735(2)
c (Å)	12.0687(8)	12.1476(5)
V (Å^3^)	352.94(3)	350.98(4)
Sr 4b (0, 1/4, 5/8)	–	–
B_iso_ (Å^2^)	0.921(2)	0.816(3)
f_occ_	1.00	1.00
Mo,M 4a (0,1/4,1/8)	–	–
B_iso_ (Å^2^)	0.813(3)	0.639(2)
Mo/M f_occ_	0.88(1)/0.12(1)	0.78(1)/0.22(1)
O 16f (x,y,z)	–	–
x	0.2378(3)	0.2388(2)
y	0.1149(4)	0.1199(3)
z	0.0432(2)	0.0449(3)
B_iso_ (Å^2^)	0.764(4)	1.00(2)
f_occ_	0.943(3)	0.963(2)
Reliability Factors	–	–
χ^2^	3.90	3.09
R_p_ (%)	3.21	2.95
R_wp_ (%)	4.31	3.73
R_exp_ (%)	2.20	2.15
R_Bragg_ (%)	6.31	3.90

**Table 2:** Selected atomic distances (Å) and angles (deg) for SrMo_1−*x*_M*_x_*O_4−δ_ from NPD at 25 °C.

Selected Atomic Distances	SrMo_0.9_Fe_0.1_O_4−δ_	SrMo_0.8_Cr_0.2_O_4−δ_
Distances (Å)	–	–
Sr–O (x4)	2.6005(2)	2.5917(2)
(x4)	2.6105(1)	2.6208(4)
<Sr–O>	2.6055	2.6062
(Mo, M)–O (x4)	1.8089(4)	1.7892(3)
Angles (°)	–	–
O–(Mo, M)–O	107.77(2)	107.89(2)
O–(Mo, M)\–O	112.74(3)	112.66(1)

## Supplementary Materials

The following are available online at https://www.mdpi.com/1996-1944/13/19/4441/s1, Figure S1: XRD patterns with Cu Kα radiation for SrMoO_4_ and SrMo_1-x_M_x_O_4-δ_ (M= Fe and Cr; x = 0.1, 0.2), characteristic of pure tetragonal scheelite phases, Table S1: Unit-cell parameters for SrMo_1-x_M_x_O_4-δ_ defined in the in tetragonal *I4_1_/a* (No 88) space group, Z = 4, from XRD at 25 °C.
