# Supplementary material for: Topotactic Oxidation of Perovskites to Novel SrMo1-xMxO4−δ (M = Fe and Cr) Deficient Scheelite-Type Oxides
Source: Materials (Basel). 2020 Oct 6;13(19):4441. doi: 10.3390/ma13194441 (PMC7578960; doi:10.3390/ma13194441)
Supplement: Supplementary file 1 [file materials-13-04441-s001.pdf]

materials

Supplementary Materials: Topotactic Oxidation of Perovskites to Novel $SrMo_{1-x}M_xO_{4-\delta}$ (M = Fe and Cr) Deficient Scheelite-Type Oxides

V. Cascos [1,2,*], R. Martínez-Coronado [1], M.T. Fernández-Díaz [3] and J.A. Alonso [1]

1 Instituto de Ciencia de Materiales de Madrid, Consejo Superior de Investigaciones Científicas, Cantoblanco, E-28049 Madrid, Spain; rmartinezcoronado@gmail.com (R.M.-C.); jaalonso@icmm.csic.es (J.A.A.)
2 Departamento de Química Inorgánica, Universidad Complutense de Madrid, E-28040 Madrid, Spain
3 Institut Laue Langevin, BP 156X, F-38042 Grenoble, France; ferndiaz@ill.fr
* Correspondence: vcascos@ucm.es; Tel.: +34-91-394-5168; Fax: +34-91-394-4352

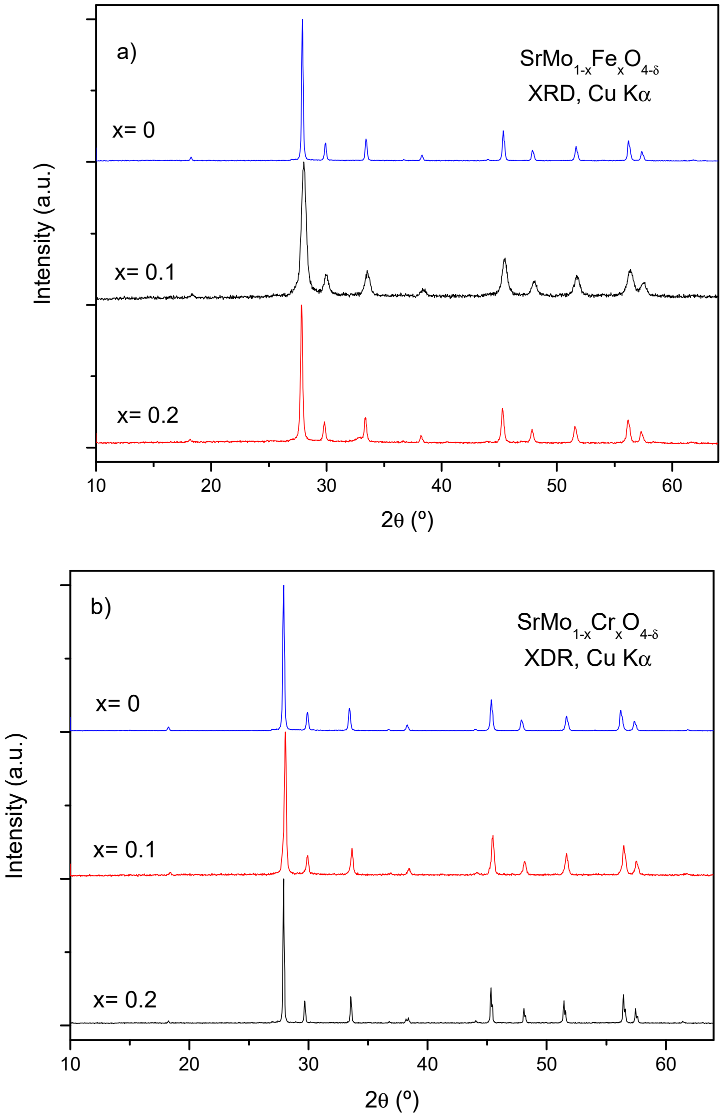

Figure S1. XRD patterns with Cu Kα radiation for (**a**) $SrMo_{1-x}Fe_xO_{4-\delta}$ (x = 0, 0.1, 0.2) and (**b**) $SrMo_{1-x}Cr_xO_{4-\delta}$ (x = 0, 0.1, 0.2), characteristic of pure tetragonal scheelite phases.

Table S1. Unit-cell parameters for $SrMo_{1-x}M_xO_{4-δ}$ defined in the in tetragonal *I4₁/a* (No 88) space group, *Z* = 4, from XRD at 25 °C.

Unit-Cell Parameters	$SrMoO_4$	$SrMo_{0.9}Fe_{0.1}O_{4-δ}$	$SrMo_{0.8}Fe_{0.2}O_{4-δ}$	$SrMo_{0.9}Cr_{0.1}O_{4-δ}$	$SrMo_{0.8}Cr_{0.2}O_{4-δ}$
a (Å)	5.3915(2)	5.3992(4)	5.4019(3)	5.3795(4)	5.3859(2)
b (Å)	5.3915(2)	5.3992(4)	5.4019(3)	5.3795(4)	5.3859(2)
c (Å)	12.0441(2)	12.0681(2)	12.0795(3)	12.1225(5)	12.1381(2)
V (Å^3)	349.84(2)	351.75(1)	352.87(2)	350.33(2)	351.06(3)

© 2020 by the authors. Licensee MDPI, Basel, Switzerland. This article is an open access article distributed under the terms and conditions of the Creative Commons Attribution (CC BY) license (http://creativecommons.org/licenses/by/4.0/).
